# Supplementary material for: Splice-Junction-Based Mapping of Alternative Isoforms in the Human Proteome
Source: Cell Rep. Author manuscript; Available in PMC 2020 Jan 15. (PMC6961840; doi:10.1016/j.celrep.2019.11.026)
Supplement: 2 [file NIHMS1546469-supplement-2.zip › DF1/PXD010154/Heart/Heart_74_LRCH4_VGGAAAVSTQAMHNLLKPGLR.pdf]

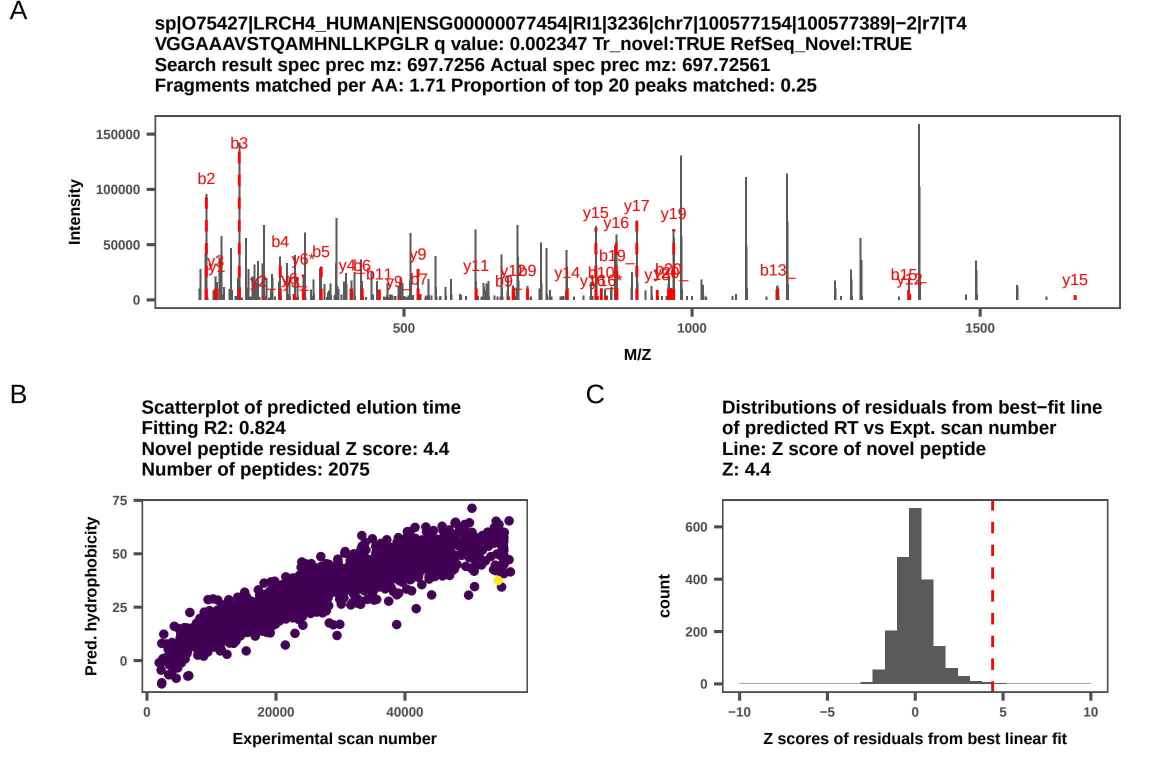
A
sp|O75427|LRCH4_HUMAN|ENSG00000077454|RI1|3236|chr7|100577154|100577389|−2|r7|T4
VGGAAAVSTQAMHNLLKPGLR q value: 0.002347 Tr_novel:TRUE RefSeq_Novel:TRUE
Search result spec prec mz: 697.7256 Actual spec prec mz: 697.72561
Fragments matched per AA: 1.71 Proportion of top 20 peaks matched: 0.25
Intensity
M/Z
B
Scatterplot of predicted elution time
Fitting R2: 0.824
Novel peptide residual Z score: 4.4
Number of peptides: 2075
Pred. hydrophobicity
Experimental scan number
C
Distributions of residuals from best−fit line
of predicted RT vs Expt. scan number
Line: Z score of novel peptide
Z: 4.4
count
Z scores of residuals from best linear fit
